# Glycolytic reprogramming in precancerous lesions of gastric cancer progression: pathogenesis and therapeutic potential

**DOI:** 10.3389/fonc.2026.1885113

**Published:** 2026-08-12

**Authors:** Siying Niu, Heyuan Sun, Zijian Wang, Qiuyu Jin, Zhiwen Sun, Dongyang Yu, Yang Zhang

**Affiliations:** 1 Graduate College, Heilongjiang University of Chinese Medicine, Harbin, China; 2 Department of Gastroenterology, The First Affiliated Hospital of Heilongjiang University of Chinese Medicine, Harbin, China; 3 Department of Gastroenterology, Hanan Branch of the Second Affiliated Hospital of Heilongjiang University of Chinese Medicine, Harbin, China

**Keywords:** glycolytic reprogramming, metabolic intervention, molecular mechanisms, pathological progression, precancerous lesions of gastric cancer

## Abstract

Precancerous lesions of gastric cancer (PLGC) represent a critical stage preceding the development of gastric cancer. Their progression to gastric cancer remains a significant challenge in clinical prevention and treatment. Recent studies have shown that glycolytic reprogramming is a dynamic and modifiable metabolic process in PLGC. It may emerge during the progression of PLGC and contribute to the pathological remodeling of the gastric mucosa. This article reviews the stage-specific characteristics, pathogenesis, and therapeutic potential of glycolytic reprogramming in PLGC. During the progression of PLGC, glycolytic activity may be activated early on, diminish during the glandular atrophy stage, and be reactivated during intestinal metaplasia (IM), dysplasia, and spasmolytic polypeptide-expressing metaplasia (SPEM). Key regulatory factors include *Helicobacter pylori* (*H. pylori)* infection, the PI3K/Akt/mTOR-HIF-1α axis, glucose uptake mediated by glucose transporters (GLUTs), microRNAs (miRNAs), the p53/TIGAR pathway, and p57. These metabolic alterations may promote PLGC progression through an imbalance between proliferation and apoptosis, lactate accumulation, and the formation of an acidic microenvironment. Intervention strategies targeting glycolytic reprogramming may offer potential approaches for metabolic intervention in PLGC. These strategies include traditional Chinese medicine formulations, natural bioactive compounds, and glycolysis inhibitors derived from gastric cancer research.

## 1 Introduction

Gastric cancer (GC) is one of the most common types of cancer worldwide (1). Under the influence of pathogenic factors such as *Helicobacter pylori* (*H. pylori*) infection, the development of GC often follows the classic Correa cascade model of pathological progression (2). The Correa cascade involves the gradual transformation of normal gastric mucosa into GC through a series of precursor lesions, including chronic inflammation, chronic atrophic gastritis (CAG), intestinal metaplasia (IM), and dysplasia (3). CAG, IM, and dysplasia are classed as PLGC (4). Early detection and effective intervention for gastric cancer have become essential strategies for its prevention.

Glycolysis affects the development and progression of GC (5). Even in the presence of sufficient oxygen, this glycolytic phenotype can meet its energy and biosynthetic needs (6). This metabolic shift supports tumor proliferation, invasion, and therapeutic resistance, highlighting the importance of glycolytic regulation in GC (7). Glycolytic reprogramming does not necessarily begin after tumor formation. Impaired mitochondrial oxidative metabolism and abnormalities in molecules associated with glycolysis can already be observed during the PLGC stage, suggesting that metabolic changes related to glycolysis may have already begun during the precancerous stage (8, 9). Therefore, interpreting PLGC from the perspective of glycolytic reprogramming helps elucidate the metabolic basis for the gradual progression of precancerous stages in the Correa cascade and provides a new theoretical basis for metabolic interventions during these stages. This review uses studies of gastric cancer metabolism as a reference and examines possible changes in glycolysis at different stages of PLGC progression. It summarizes the related regulatory mechanisms, including the inducing role of H. pylori. It also discusses the value of targeting glycolysis for early metabolic intervention in PLGC. Finally, the review examines the limits of current evidence and the main unanswered questions to guide future research.

## 2 Research methods

This article researched studies published from January 2016 to May 2026 in PubMed and Web of Science databases. The search terms included “gastric precancerous lesions,” “precancerous lesions of gastric cancer,” “chronic atrophic gastritis,” “intestinal metaplasia,” “gastric dysplasia,” “SPEM,” “glycolysis,” “glycolytic reprogramming,” “glucose metabolism,” “lactate,” and “Warburg effect.” Additional searches used the names of the main pathways, molecules, and treatments discussed in this review, including Helicobacter pylori, HIF-1α, PI3K/Akt/mTOR, glucose transporters, microRNAs, p53/TIGAR, p57, traditional Chinese medicine, and natural compounds. The detailed search strategies are provided in **Supplementary Data 1**. We prioritized original studies that directly examined PLGC. When direct PLGC evidence was limited, we included studies on gastric cancer or other experimental models as indirect evidence. The evidence supporting glycolytic alterations and related mechanisms in PLGC is summarized in **Supplementary Table 1**. Relevant reviews and the reference lists of included studies were also checked. Older key studies were included when needed to explain basic mechanisms. Classic reviews and key studies published before 2016 were also included to explain cancer metabolism, metabolic reprogramming, and the Warburg effect.

## 3 From physiological glycolysis to GC-associated glycolytic reprogramming

Cells derive energy primarily from OXPHOS in the mitochondria and aerobic glycolysis (10, 11). Glycolysis is the process by which glucose is converted into pyruvate through a series of enzymatic reactions in the cytoplasm, while producing small amounts of ATP and NADH (12). Under conditions of sufficient oxygen, pyruvate typically enters the mitochondria to participate in the citric acid cycle and oxidative phosphorylation (OXPHOS) (13, 14). However, under hypoxic or metabolic stress conditions, pyruvate can be reduced to lactate by lactate dehydrogenase to regenerate NAD^+^ and sustain glycolysis (15). Therefore, glycolysis itself is not an abnormal process, but rather an essential mechanism by which cells maintain their basic energy metabolism. Glycolytic reprogramming is regulated by cancer-related signaling and changes in the tumor microenvironment (16, 17).

Unlike normal glycolysis, tumor-associated glycolytic reprogramming is primarily characterized by increased glucose uptake and lactate production even under conditions of relatively sufficient oxygen, known as the Warburg effect (18, 19). Glycolysis occurs rapidly and is less efficient at producing ATP than OXPHOS, allowing cells to obtain energy quickly. Meanwhile, glycolysis and its related metabolic branches provide metabolic substrates for the biosynthesis of nucleotides, amino acids, and lipids. These metabolic processes help sustain the energy and biosynthetic needs of abnormally proliferating cells and supporting malignant phenotypes such as tumor cell proliferation, growth, invasion and metastasis (20). Lactate produced by glycolysis is exported from cells, acidifying the local metabolic microenvironment. This may lead to immune evasion, angiogenesis, invasion, and metastasis (21–24).

## 4 Characteristics of glycolytic reprogramming at different stages of PLGC

**Figure 1:**
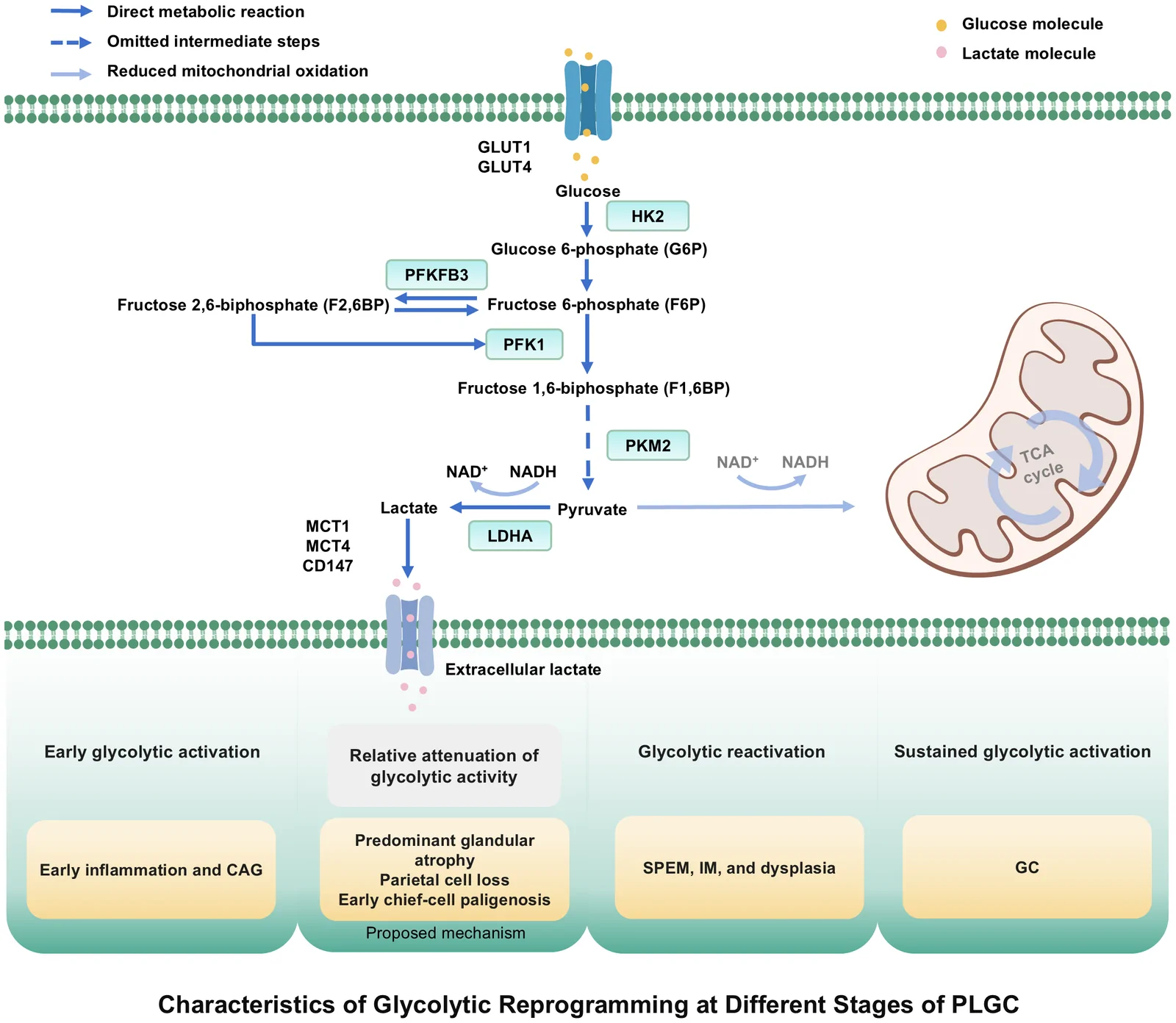
Characteristics of glycolytic reprogramming at different stages of PLGC: Glycolytic reprogramming is activated during early inflammation and CAG. It may be relatively attenuated during predominant glandular atrophy, parietal cell loss, and early chief-cell paligenosis. It may be reactivated during SPEM, IM, and dysplasia. Sustained glycolytic reprogramming may occur in GC. The core glycolytic pathway remains unchanged, but its relative activity may vary across pathological stages. These stage-specific changes represent a proposed qualitative pattern rather than a quantitative comparison.

Available evidence suggests that glycolytic activity changes across different stages of PLGC. It shows a dynamic pattern characterized by early activation, a phased decline, and subsequent reactivation. Studies reported changes in HIF-1α, molecules involved in glycolysis, glucose, pyruvate, and lactate during the CAG stage. These findings suggest changes in glucose metabolism at this stage. Notably, this process does not continue to increase as the disease progresses. During the stage characterized primarily by atrophy and reduced mucosal intrinsic glands, glycolytic activity may be relatively diminished. This may be related to parietal cell atrophy, during which chief cells may enter the initial phase of paligenosis, resulting in diminished energy requirements (25, 26). As the lesion progresses to IM and dysplasia, the proliferative activity of gastric mucosal epithelial cells increases, thereby increasing their energy and biosynthetic requirements. The expression of glucose transporter and HIF-1α regulatory molecules was elevated, consistent with further enhancement of glycolytic activity. These metabolic changes may contribute to the continued proliferation of abnormal epithelial cells and disease progression (27). In particular, SPEM serves as an important precursor lesion in IM and a critical step in the malignant progression of the gastric mucosa. A shift from oxidative metabolism toward glycolysis has been demonstrated in SPEM. This conclusion is supported at three complementary levels, including altered expression of metabolic molecules, increased glycolytic flux, and changes in glycolysis-related metabolites (28). However, this pattern occurs in PLGC still needs to be determined by measuring glycolytic flux at different stages and by studies that follow patients over time. The evidence sources and levels for the major glycolysis-related findings in PLGC are summarized in **Supplementary Table 1**. This proposed stage-specific pattern of glycolytic reprogramming is illustrated in **Figure 1**.

## 5 Pathogenic mechanisms of glycolytic reprogramming in PLGC progression

**Figure 2:**
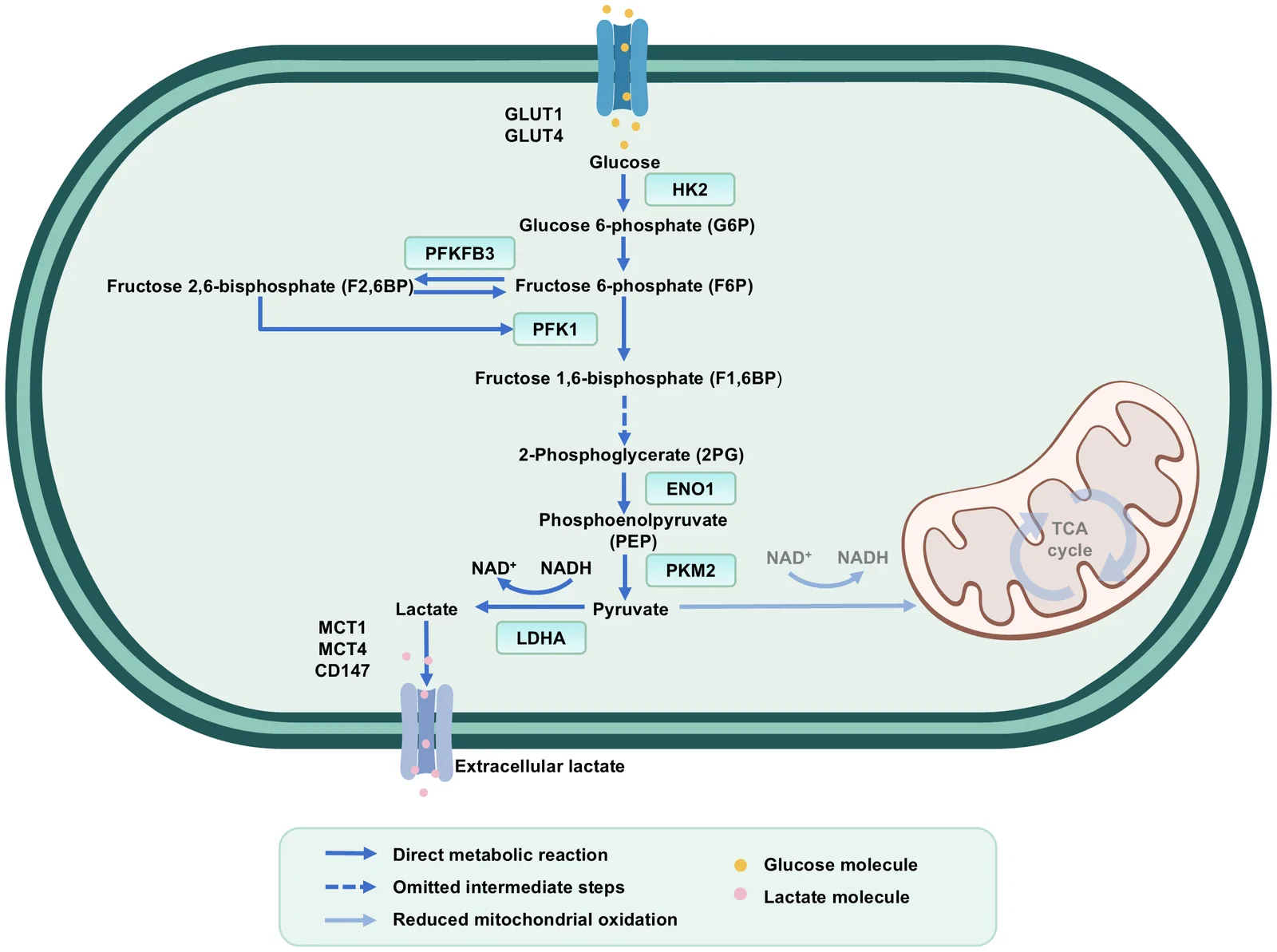
Metabolic pathway for glycolysis in PLGC: In PLGC, glucose uptake via GLUT1 and GLUT4 is increased. This glucose then enters an enhanced glycolytic pathway mediated by key enzymes such as HK2, PFKFB3, PFK1, ENO1, PKM2, and LDHA. Under conditions of glycolytic reprogramming, pyruvate is preferentially converted to lactate by LDHA, accompanied by NAD^+^ regeneration. This sustains continuous glycolysis and promotes lactate accumulation. Concurrently, the metabolic flux of pyruvate entering the mitochondrial tricarboxylic acid (TCA) cycle is relatively reduced. Excess lactate is exported through MCT1 and MCT4 with support from CD147. This process contributes to remodeling of the local metabolic microenvironment in PLGC.

In PLGC, glycolytic reprogramming links upstream signaling activation with increased glucose uptake, altered glycolytic flux, enhanced pyruvate-to-lactate conversion, and dysregulated lactate export. HIF-1α is central to this regulatory network (29), as it regulates several glycolysis-related molecules, including GLUT1 and GLUT4, HK2, PFKFB3, ENO1, PKM2, and LDHA. This promotes glucose uptake into cells, enhances key steps in glycolysis, increases lactate production, and drives changes in the local metabolic microenvironment of the diseased gastric mucosa (30, 31). Among these, GLUT1 and GLUT4 are primarily involved in the transmembrane uptake of glucose; HK2 initiates glucose phosphorylation, a key step in the early stages of glycolysis (32, 33); PFKFB3 increases the flux of glycolysis by enhancing PFK1 activity; ENO1 and PKM2 participate in late-stage glycolytic reactions and promote pyruvate production (34); LDHA converts pyruvate into lactate, further linking enhanced glycolysis to lactate accumulation. The generated lactate can be excreted via MCT1 and MCT4 and their partner protein CD147, contributing to the maintenance of acid-base homeostasis and the remodeling of the local microenvironment **Figure 2** (35, 36).

**Figure 3:**
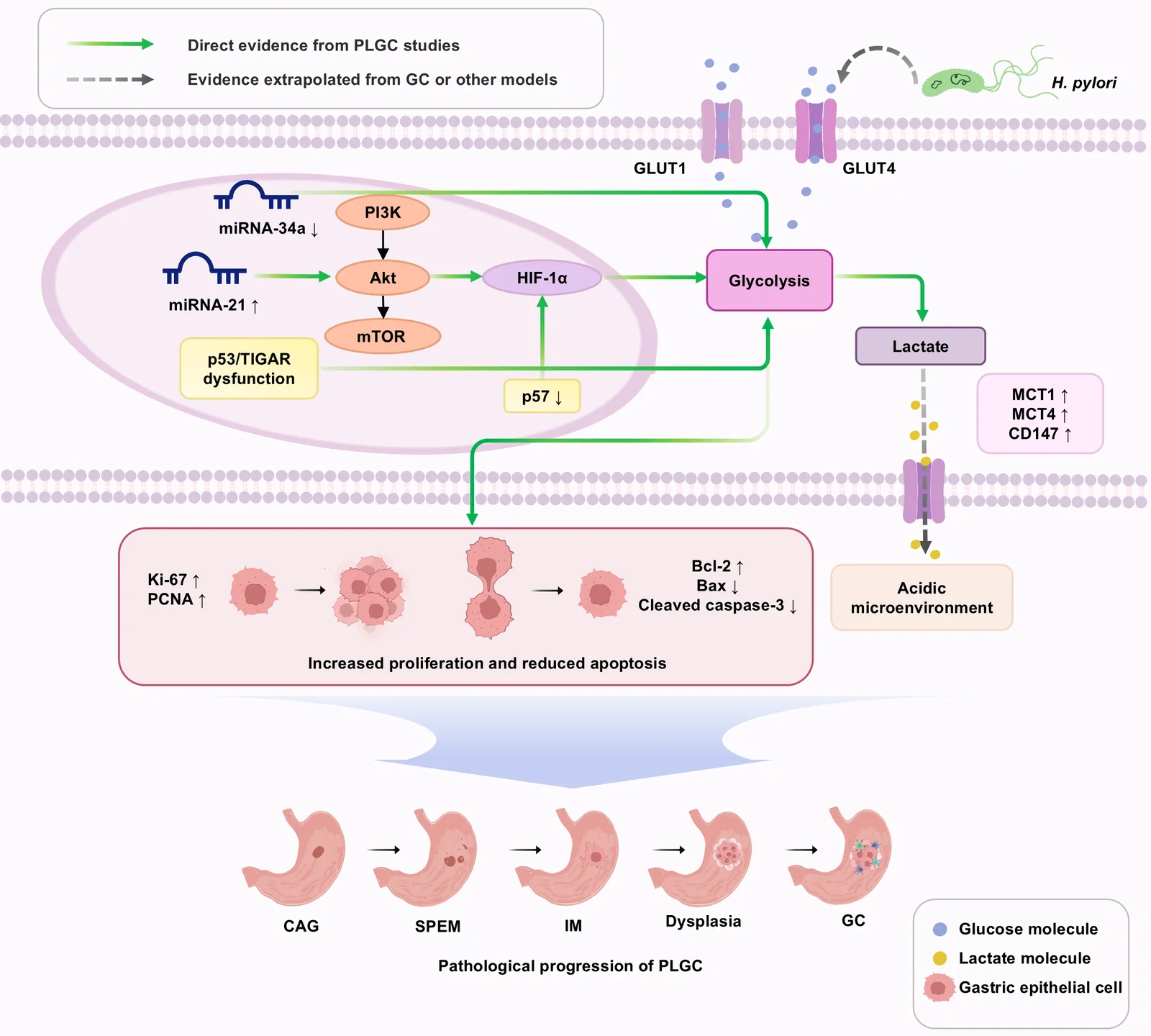
Molecular mechanisms and pathological effects of glycolysis in PLGC: The reprogramming of glycolysis in PLGC is regulated by multiple upstream factors, including *H. pylori* infection, enhanced glucose uptake mediated by GLUT1 and GLUT4, activation of the PI3K/Akt/mTOR-HIF-1α axis, dysregulation of miRNA-34a and miRNA-21 expression, dysfunction of the p53/TIGAR pathway, and reduced p57 expression. These regulatory events collectively promote glycolytic activation and increased lactate production. Lactate export mediated by MCT1 and MCT4, with the support of CD147, contributes to lactate accumulation and the formation of an acidic metabolic microenvironment. This process, in conjunction with abnormal proliferation of gastric mucosal epithelium and suppressed apoptosis, may contribute to the progression of chronic atrophic gastritis, SPEM, IM, dysplasia, and GC. Solid arrows represent direct PLGC evidence, whereas dashed arrows represent indirect evidence extrapolated from gastric cancer or other experimental models.

Glycolytic reprogramming in PLGC is also subject to multiple levels of upstream regulation. *H. pylori* infection provides an inducing background for glycolytic reprogramming. The PI3K/Akt/mTOR-HIF-1α axis, microRNAs, the p53/TIGAR tumor-suppressing metabolic axis, and p57-related regulatory mechanisms further influence the expression and activity of key molecules in glycolysis. The following sections will describe the molecular mechanisms underlying glycolytic reprogramming in PLGC, focusing on these regulatory levels **Figure 3**.

### 5.1 *H. pylori* infection is an upstream inducer of glycolytic reprogramming

*H. pylori* infection is a key early event in the development of PLGC (37–39). Its induction of chronic inflammation, oxidative stress, the HIF-1α response, and mitochondrial dysfunction collectively constitute the upstream inducing background for glycolytic reprogramming during the PLGC phase. Glycolytic reprogramming is a metabolic adaptation that occurs under conditions of mitochondrial dysfunction, impaired electron transport chain (ETC) activity, and microenvironmental stressors such as hypoxia and nutrient competition (40, 41). Research shows that *H. pylori* infection can upregulate Lon protease 1 (LONP1) and enhance glycolysis in gastric epithelial cells. LONP1 is involved in mitochondrial protein quality control and the mitochondrial stress response. Its upregulation suggests that *H. pylori* may disrupt mitochondrial homeostasis and shift cellular metabolism toward glycolysis. This evidence demonstrates that *H. pylori* infection is associated with mitochondrial stress and glycolytic reprogramming (42). Additionally, *H. pylori* can induce the expression and nuclear translocation of HIF-1α via the PI3K/mTOR pathway (43). HIF-1α, in turn, regulates the expression of glycolysis-related molecules, thereby promoting the activation of glycolytic reprogramming (44).

*H. pylori* virulence factors can further exacerbate this metabolic reprogramming. CagA can influence glucose metabolism-related molecules such as PKM2 and PDK1 and promotes ROS accumulation and HIF-1α stabilization by inhibiting the mitochondrial deacetylase SIRT3 (45). VacA can induce mitochondrial dysfunction, mitochondrial fission, and a reduction in mitochondrial DNA copy number (46–48). This suggests that *H. pylori* may drive compensatory enhancement of glycolysis by disrupting mitochondrial homeostasis. Further studies on GC cells have shown that CagA overexpression enhances glucose uptake and lactate production, upregulates p-Akt, HK2, and LDHA, and is associated with 5-FU resistance; inhibition of glycolysis or the Akt pathway attenuates this phenotype (49).

Therefore, in the early stages of the Correa cascade, *H. pylori* may contribute to the reprogramming of glycolysis during the PLGC phase through inflammatory responses, mitochondrial damage, and HIF-1α-mediated metabolic regulation. However, most of the current evidence still comes from gastric epithelial cell or GC cell models. Whether the mechanisms described above have already led to a stable increase in glucose flux at the PLGC stage requires further confirmation through animal models, human gastric mucosal samples, and flux studies.

### 5.2 The PI3K/Akt/mTOR-HIF-1α-mediated activation pathway of classical glycolysis

Within the regulatory network of glycolysis, the PI3K/Akt/mTOR-HIF-1α axis is one of the classical signaling pathways and may also participate in glycolytic reprogramming during PLGC. The PI3K/Akt signaling pathway plays a crucial role in cell proliferation, survival, autophagy, and metabolic regulation (50, 51). HIF-1α, a key transcription factor in glycolysis, plays a central role in the hypoxic response and is also associated with malignant biological processes such as angiogenesis, invasion, and metastasis (52). Previous studies have shown that activation of the PI3K/Akt signaling pathway enhances mTOR activity, thereby promoting the expression and stabilization of HIF-1α under normoxic conditions. This, in turn, induces the expression of glycolysis-related molecules, increases glycolytic activity and lactate production, and drives metabolic reprogramming during PLGC (53). At the same time, its activation is often accompanied by persistent inflammation, glandular atrophy, IM, and abnormal epithelial proliferation, suggesting an intrinsic link between enhanced glycolysis and the progression of the disease (54). Inhibition of this pathway is associated with downregulation of molecules involved in glycolysis and alleviation of pathological damage, further suggesting that it may constitute a key metabolic driver of PLGC progression.

### 5.3 Abnormal expression of GLUT1 and GLUT4 in PLGC

During the PLGC phase, abnormalities in glucose metabolism do not necessarily manifest initially as significant changes in typical glycolytic enzymes or metabolic end products. Altered glucose uptake represents an important component of metabolic reprogramming during lesion progression (55). In PLGC rat models and human IM, dysplasia, and early GC tissues, increased expression of GLUT1, GLUT3, and GLUT4 has been reported. The GLUT family, as key molecules mediating the transmembrane transport of glucose in cells, is believed to play a fundamental role in metabolic reprogramming when its expression is abnormal.

In GC, overexpression of GLUT1 or GLUT4 enhances glucose uptake and increases the activity of glycolysis-related enzymes such as HK2 and LDHA. By increasing glucose uptake into cells, GLUT1 or GLUT4 overexpression can increase lactate and ATP production, thereby supporting tumor cell growth and survival (56–58). In the PLGC rat model, the expression of GLUT1 and GLUT4 is also elevated. In human IM, dysplasia, and early-stage GC tissues, GLUT1, GLUT3, and GLUT4 also show a similar upward trend, with GLUT1 and GLUT4 having a closer relation to disease progression. Further intervention revealed that downregulation of GLUT1 and GLUT4 expression can improve changes related to IM, dysplasia, and angiogenesis in the gastric mucosa (59). However, direct evidence confirming an increase in glycolytic flux in PLGC remains limited at this time.

### 5.4 Regulation of glycolysis mediated by microRNAs

miRNAs are a class of endogenous non-coding single-stranded small RNAs that regulate the expression of target genes at the post-transcriptional level and are involved in the upstream regulation of glycolytic reprogramming in PLGC. Among these, miRNA-34a and miRNA-21 are particularly representative; both are closely associated with GC-related biological processes such as cell proliferation, differentiation, invasion, and metastasis (60). miRNA-34a is a tumor suppressor that can inhibit the growth, invasion, and metastasis of GC cells (61, 62). In an MNNG (N-methyl-N’-nitro-N-nitrosoguanidine)-induced PLGC model, its expression is downregulated, accompanied by elevated LDHA levels. This suggests that its absence may weaken the inhibitory effect on key enzymes of glycolysis, thereby promoting enhanced glycolysis. In addition, miRNA-34a may also participate in glycolytic reprogramming by regulating PI3K/Akt/mTOR-related signaling pathways (63).

Unlike miRNA-34a, miRNA-21 is predominantly oncogenic. It inhibits phosphatase and tensin homolog, thereby activating the PI3K/Akt pathway and leading to increased proliferation, cancer cell survival, and Bcl-2 expression (64). In the PLGC model of Atp4a^−/−^ mice, miRNA-21 expression is increased. This increase is accompanied by the upregulation of HK2 and LDHA, increased Bcl-2, and decreased caspase-3 levels. Rg3 treatment reverses these changes, possibly through PI3K/Akt-related signaling. These findings suggest that miRNA-21 may be associated with glycolytic reprogramming and apoptosis regulation during PLGC (65). However, current research on the role of miRNAs in regulating glycolytic reprogramming in PLGC remains limited. Their causal roles in glycolysis during PLGC still need to be verified through functional experiments.

### 5.5 p53/TIGAR mediates glycolytic reprogramming

The role of the p53/TIGAR axis in PLGC-associated glycolytic reprogramming is influenced by the functional status of p53, TIGAR expression, and the stage of the disease. Under physiological conditions, wild-type p53, as a tumor suppressor, restricts malignant transformation by regulating cell cycle arrest, DNA repair, apoptosis, senescence, and metabolic homeostasis. Regarding glucose metabolism, functional p53 supports OXPHOS and limits excessive glycolysis by inhibiting glycolysis, downregulating Akt/mTOR signaling, and transcriptionally activating TIGAR (66, 67). TIGAR inhibits PFK1 activity and redirects glucose metabolism toward the pentose phosphate pathway, thereby contributing to NADPH production and the maintenance of ROS homeostasis (68).

However, the accumulation of p53 in precancerous or cancerous lesions does not necessarily indicate the functional activation of wild-type p53. TP53 mutations, impaired p53 function, or stress-induced stabilization can all lead to different biological outcomes. Some mutant forms of p53 may even acquire functions that promote glycolysis, proliferation, and metabolic adaptation (69, 70). In the PLGC model, p53-related metabolic regulation exhibits complexity. In MNNG-induced PLGC, p53 accumulation is accompanied by decreased TIGAR and increased LDHA. This suggests that the p53/TIGAR metabolic “braking” mechanism may be impaired (71). In contrast, TIGAR expression was elevated in another MNNG-induced PLGC model and in human PLGC samples. Following GRg3 intervention, TIGAR, G6PDH, NADP, and GSH levels decreased, while ROS levels further increased, apoptosis was enhanced, and PCNA expression decreased. This suggests that TIGAR may help abnormal gastric mucosal epithelial cells maintain redox homeostasis, enabling them to evade apoptosis and sustain abnormal proliferation under conditions of elevated ROS (72). In the Atp4a^−/−^ mouse model, strong p53 expression was accompanied by elevated Ki-67 labeling, activation of the PI3K/Akt/mTOR/HIF-1α pathway, and upregulation of LDHA. This suggests a possible link between p53-related stress responses, proliferative reprogramming, and glycolytic activation (53).

Therefore, the p53/TIGAR axis in PLGC is not a unidirectional pathway that inhibits glycolysis. Its function may depend on the functional state of p53, the expression pattern of TIGAR, the stage of the disease, and the cellular redox state. The role of this pathway in metabolism needs to be interpreted in conjunction with p53 status, TIGAR expression, and disease stage.

### 5.6 p57 mediates glycolytic reprogramming

p57 is a candidate tumor suppressor gene in the cyclin-dependent kinase-interacting protein/kinase inhibitory protein family. It suppresses tumorigenesis by regulating key processes such as cancer cell proliferation, migration, and aerobic glycolysis (73). Elevated p57 levels may be accompanied by reduced aerobic glycolysis and enhanced mitochondrial OXPHOS (74). In the gastric mucosa, p57 is also a key molecule involved in maintaining the homeostasis of chief cells. It is considered a critical molecular switch that regulates the “reserve stem cell” state of chief cells. Following damage to the gastric mucosa, p57 expression in chief cells decreases, causing them to exit their quiescent state and enter a proliferative response (75, 76). In the SPEM model, downregulation of p57 is not only associated with the loss of primary cell characteristics and abnormal proliferation but is also closely linked to enhanced glycolytic reprogramming. This is manifested by the upregulation of HIF-1α, LDHA, and PKM2 (28). Therefore, p57 may serve as a key link between cellular homeostasis disruption and the activation of glycolytic reprogramming, and may be involved in the pathological progression of SPEM. However, the dynamic expression of p57 during different stages of PLGC and its relationship to the transition of primary cell fate remain to be clarified.

## 6 The pathological role of glycolytic reprogramming in PLGC

### 6.1 Imbalance between cell proliferation and apoptosis and the evolution of pathological phenotypes

Normally, balanced proliferation and apoptosis maintain the homeostasis of the gastric mucosal epithelium (77, 78). *H. pylori* infection, chronic inflammation, and persistent damage can disrupt this balance, making abnormal epithelial cells more likely to proliferate and less sensitive to apoptosis (79–81). Thus, abnormal cells may gradually accumulate, driving the gastric mucosa to progress further along the Correa cascade (82, 83). Ki-67 and PCNA reflect abnormal proliferation (84, 85), while the Bax/Bcl-2 ratio and cleaved caspase-3 reflect the activation of the mitochondrial apoptotic pathway (86, 87). Enhanced glycolysis can meet the energy and biosynthetic demands of rapidly proliferating abnormal epithelial cells and influence cell proliferation and apoptosis. In the PLGC model, both the modified Zuojin Pill and the modified Zuojin formula reduced levels of HIF-1α-related glycolytic signaling. These changes were accompanied by decreased expression of Ki-67 and PCNA, and elevated levels of apoptotic markers including Bax, the Bax/Bcl-2 ratio, and cleaved caspase-3 (27, 88). Overall, glycolytic reprogramming may contribute to the pathological progression of PLGC by influencing the aforementioned molecules associated with proliferation and apoptosis. While existing evidence suggests that glycolysis is involved in the proliferation-apoptosis imbalance, the causal relationship between the two requires further verification.

### 6.2 Lactate accumulation and microenvironmental remodeling

Lactate was once regarded as a metabolic waste product, but it is now recognized as an active participant in cancer biology (89). It functions both as a signaling molecule and as a source of energy (90, 91). In tumor cells with enhanced glycolysis, upregulation of LDHA promotes the conversion of pyruvate to lactate, leading to increased lactate production. To maintain a state of high glycolysis and intracellular acid-base homeostasis, excess lactate must be continuously exported via the monocarboxylate transporters MCT1 and MCT4. Their membrane localization and functional stability depend on the support of the chaperone protein CD147 (92, 93). These findings suggest that lactate accumulation and the resulting acidic microenvironment may provide favorable local metabolic conditions for the progression of PLGC to higher-grade lesions. Studies have shown that high expression of CD147 and MCT4 in PLGC epithelium suggests that the lactate efflux system may be activated. Abnormal regulation of the p53/miRNA-34a/LDHA and p53/TIGAR pathways in PLGC is accompanied by upregulation of HIF-1α, CD147, MCT1, and MCT4. This promotes lactate transport and disease progression (71, 94). Together, these observations indicate that lactate efflux mediated by MCT1, MCT4, and CD147 may already be involved in PLGC progression. However, current research remains largely limited to changes in the expression of molecules such as MCT1, MCT4, and CD147 expression, and there is still a lack of direct evidence regarding changes in lactate concentration, extracellular acidification status, and the dynamics of the local metabolic microenvironment. Future studies will need to integrate metabolomics, lactate flux assays, and spatial multi-omics technologies to further elucidate the role of lactate efflux in the progression of PLGC and its potential for therapeutic intervention.

## 7 Preclinical evidence for interventions that target glycolysis in PLGC

### 7.1 The effects of TCM formulations and natural bioactive compounds on glycolysis

TCM is characterized by its multi-component, multi-target, and multi-stage regulatory effects in PLGC interventions. Current research suggests that the regulation of glycolytic reprogramming by TCM formulations and natural bioactive compounds primarily occurs at three levels. First, it inhibits the HIF-1α-mediated glycolysis activation pathway, downregulates key molecules such as HK2, PFKFB3, PKM2, and LDHA, and improves glucose utilization, lactate production, and pathological damage to the gastric mucosa. Second, it regulates glucose uptake and lactate transport processes, including downregulating molecules such as GLUT1/GLUT4, MCT1/MCT4, and CD147, thereby potentially attenuating glucose-dependent metabolic adaptation and lactate-related microenvironmental changes. Third, it influences upstream regulatory nodes such as miRNA-34a/LDHA, miRNA-21/PI3K/Akt, p53/TIGAR, and p57, participating in abnormal proliferation, apoptosis imbalance, and the remodeling of glucose metabolism networks. Overall, interventions using TCM to reprogram glycolysis in PLGC are more consistent with the characteristics of “multi-node network regulation”; representative studies are listed in **Table 1**. The detailed botanical compositions of the TCM formulations listed in **Table 1** are provided in **Supplementary Table 2**. However, the current evidence is primarily based on animal and cell studies. Most studies mainly assessed the expression of glycolysis-related molecules rather than direct metabolic function. Metabolic flux analysis, target rescue experiments, human validation, and long-term pathological outcomes remain limited. Implications of Glycolysis Inhibitors for Translational Research on PLGC Intervention.

**Table 1:** Evidence for Glycolytic Alterations and Mechanisms in PLGC.

Intervention	Composition or source	Experimental model and sample size	Dose and duration	Main mechanism	Metabolic endpoints	Pathological and biological endpoints	Main findings	Evidence level and key limitations	Reference
Modified Zuojin pill, SQQT TCM formula	*Astragalus mongholicus, Citrus reticulata, Panax notoginseng, Coptis chinensis, Tetradium ruticarpum, Glycyrrhiza glabra*	MNNG- induced PLGC rats (n = 10/group)	0.63, 1.26, or 2.51 g/kg/day for 10 weeks	HIF-1α↓	Glucose, pyruvate, lactate, LDH, HIF-1α, GLUT1, GLUT4, HK2, PFKFB3, PKM2, and LDHA	intestinal metaplasia, dysplasia, inflammation, PCNA, Ki-67, cleaved caspase 3, apoptosis, and cell migration	Improved gastric mucosal pathology; reduced glycolysis-related metabolites and proteins; inhibited proliferation; promoted apoptosis	E2; no direct glycolytic flux measurement no human validation	(27)
		MNNG- treated MC cells	10% SQQT containing serum						
Modified Zuojin formula, MZJF TCM formula	*Astragalus mongholicus, Citrus reticulata, Panax notoginseng, Coptis chinensis, Tetradium ruticarpum, Glycyrrhiza glabra*	MNNG- induced CAG rats (n = 10/group)	1.26 g/kg/day for 12 weeks	HIF-1α↓	Glucose, pyruvate, lactate, HIF-1α, GLUT1, GLUT4, HK2, PFKFB3, PKM2, and LDHA	glandular atrophy, intestinal metaplasia, dysplasia, inflammatory factors, parietal and chief cell ultrastructure, PG I, PG II, PGR, Bax, Bcl-2, cleaved caspase-3	Improved gastric mucosal pathology and cellular ultrastructure; reduced inflammation and glycolysis-related changes; promoted apoptosis	E2; single-dose design network pharmacology and docking are predictive no direct glycolytic flux measurement	(88)
Xiaojianzhong decoction, XJZ TCM formula	Maltose, Cinnamomum cassia, Paeonia lactiflora, ginger, Glycyrrhiza species, jujube	MNNG- induced PLGC rats (n = 5/group)	1.875, 3.75, or 7.50 g/kg/day for 4 weeks	PI3K/AKT/mTOR↑ p53/AMPK/ULK1↓	HIF-1α, CD147, MCT1, MCT4, SIRT6, ULK1, PI3K, AKT, mTOR, p53, and AMPK	intestinal metaplasia, dysplasia, CDX2, Ki 67, autophagosomes, Bnip3, Beclin 1, LC3II, p62, body weight, oxidative stress, and liver and kidney indicators	Improved gastric mucosal lesions; reduced hypoxia and lactate transport markers; inhibited excessive autophagy; reduced intestinal metaplasia and dysplasia	E2; Glycolysis inferred from related markers no direct glycolytic flux measurement or rescue experiment	(94)
Weipixiao decoction, WPX TCM formula	*Astragalus membranaceus, Pseudostellaria heterophylla, Atractylodes macrocephala, Salvia miltiorrhiza, Oldenlandia diffusa* *Salvia miltiorrhiza*	MNNG- induced PLGC rats (n = 10/group)	9.9 or 19.8 g/kg/day for 10 weeks	miRNA-34a/LDHA↑ PI3K/AKT/mTOR/HIF-1α↓	miR-34a, LDHA, CD147, MCT4, HIF-1α, PI3K, AKT, and mTOR	intestinal metaplasia, dysplasia, and gastric epithelial morphology	Improved intestinal metaplasia and dysplasia; increased miR-34a; reduced glycolysis and lactate transport related molecules	E2; glycolysis inferred from related markers no direct glycolytic flux measurement or target validation	(63)
Pristimerin Natural bioactive compound	*Celastrus orbiculatus*	HDT-induced SPEM mice (n = 6/group)	0.05, 0.1, and 0.2 mg/kg/day for 21 days	p57↑	OCR, ECAR, glycolytic rate, glycolytic capacity, pyruvate, lactate, HIF-1α, LDHA, PKM2, p57	oxyntic atrophy, TFF2, MUC6, WFDC2, GIF, H⁺/K⁺-ATPase, Mist1, Ki67, Lgr5, Troy	Improved SPEM pathology; restored oxyntic glands; suppressed glycolysis; reduced SPEM and stem cell markers; upregulated p57	E2; small sample size no human validation	(28)
		TAM-treated GES-1 cells (n = 5)	0.1, 0.2, and 0.4 μmol/L for 24 h in cells						
		MNNG and H. pylori-induced gastric organoids (n = 5)	0.2 μmol/L for 24 h in organoids						
Hederagenin Natural bioactive compound	Pentacyclic triterpenoid from medicinal plants	MNNG-induced CAG rats (n = 6/group)	10, 20, and 40 mg/kg/day for 4 weeks	mTOR/HIF-1α↓	Glucose uptake, lactate, HK II, PKM2, ENO1, LDHA, HIF-1α, p-mTOR	glandular atrophy, intestinal metaplasia, PG I, PG II, G-17, IL-6, proliferation, migration, invasion	Improved gastric pathology; reduced intestinal metaplasia and inflammation; suppressed glycolytic activity; inhibited malignant phenotypes	E2; no Seahorse assay or human validation	(54)
		MNNG-transformed MC cells (n ≥ 3)	5, 10, and 20 μmol/L for 24 h in cells						
		mTOR siRNA validation							
Ginsenoside Rg3 Natural bioactive compound	*Panax ginseng*	Atp4a⁻/⁻ PLGC mice (n = 10/group)	5 and 10 mg/kg/day for 10 weeks	miRNA-21↓ PI3K/AKT/mTOR/HIF-1α↓	PI3K, p-AKT, mTOR, HIF-1α, HK II, LDHA, miRNA-21	intestinal metaplasia, dysplasia, Bax, Bcl-2, caspase-3	Improved PLGC pathology; reduced glycolysis-related proteins; downregulated miRNA-21; promoted apoptosis	E2; glycolysis inferred from molecular markers; no functional metabolic assessment possible Atp4a deletion-related effects	(65)
Astragaloside IV Natural bioactive compound	*Astragalus membranaceus*	MNNG-induced PLGC rats (n = 3 /group)	50 and 100 mg/kg/day for 10 weeks	p53/miRNA-34a/LDHA↑ p53/TIGAR↑	LDHA, p53, TIGAR, MCT1, MCT4, CD147, HIF-1α, miRNA-34a	intestinal metaplasia, dysplasia, gastric epithelial ultrastructure	Improved PLGC pathology; reduced glycolysis-related molecules; restored TIGAR; increased p53 and miRNA-34a	E2; glycolysis inferred from molecular markers no glucose uptake, lactate, or glycolytic flux assessment	(71)

Evidence level: E1, direct evidence from human PLGC tissues or clinical samples; E2, evidence from PLGC-relevant animal models, gastric organoids, or non-malignant gastric epithelial models; E3, indirect evidence from GC tissues or experimental GC models; E4, extrapolated mechanistic evidence from other cancers, non-gastric systems, or foundational studies. The tier reflects relevance to PLGC rather than formal methodological quality. Reviews, guidelines, expert opinions, and meta-analyses were not assigned evidence tiers and were excluded from this study-level table. Changes in gene or protein expression were not considered equivalent to direct changes in glycolytic flux.

### 7.2 Implications of glycolysis inhibitors for translational research on PLGC intervention

Research on GC glycolysis inhibitors not only provides therapeutic strategies for GC but also offers candidate targets and directions for experimental validation for “precancerous intervention” at the PLGC stage. 2-Deoxy-D-glucose (2-DG) is a classic glucose analog and glycolysis inhibitor that enters cells via glucose transporters and is phosphorylated by hexokinase to form 2-DG-6-phosphate. Because 2-DG-6-phosphate cannot proceed to subsequent glycolytic reactions, its accumulation within the cell competitively inhibits glucose metabolism, thereby reducing glycolytic flux, ATP production, and lactate generation (95, 96). In addition to inhibiting tumor cell proliferation by reducing glycolytic flux and energy supply, it may also affect glycolysis-dependent pro-migratory signaling, thereby reducing the metastatic potential of tumor cells (97). Unlike 2-DG, which acts on the early steps of glycolysis, the PFKFB3 inhibitor PFK15 more specifically targets the regulation of glycolytic flux. PFKFB3 inhibitor PFK15 is one of the small-molecule anti-glycolysis inhibitors in GC; it reduces the production of fructose-2,6-bisphosphate (F2,6BP) and glucose uptake. Consequently, it induces G0/G1 phase arrest and mitochondrial apoptosis, and inhibits GC cell invasion and xenograft tumor growth (98, 99). Research on GC glycolysis inhibitors suggests that inhibiting glycolytic flux can limit the invasive phenotype of pathological cells by reducing energy supply and lactate production, restoring the balance between proliferation and apoptosis, and attenuating pro-migratory signals. Studies in GC identify HK2 and PFKFB3 as possible targets for PLGC research. This strategy still needs to be tested in PLGC-specific systems, including animal models and gastric organoids, together with metabolic flux analysis and long-term carcinogenesis endpoints to assess efficacy, safety, and pathological reversal. Only by clearly defining the therapeutic window and intervention boundaries in PLGC-specific models can research on GC glycolysis inhibitors be effectively translated into metabolic blockade strategies for the precancerous stage. Most interventions have only been tested in animal or cell models. Their dose, target specificity, metabolic toxicity, long-term safety, and effects on pathological reversal remain unclear.

### 7.3 Translational implications of glycolysis-related therapeutic resistance in GC for PLGC

Studies of glycolysis-related drug resistance in GC provide a basis for examining whether similar metabolic adaptation also occurs in PLGC. In many cancer types, cancer cells may contribute to therapeutic resistance through enhanced glycolysis (100, 101). This represents a major challenge in cancer treatment and management. Trastuzumab is an important monoclonal antibody used in the standard treatment of advanced HER2-positive GC (102). The main challenge is that most patients develop resistance to it (103, 104). Studies have shown that thioridazine can directly bind to and downregulate Skp2, thereby further inhibiting the Skp2/Akt/mTOR/GLUT1 signaling pathway, reducing glucose uptake and lactate production. This enhances the antitumor activity of trastuzumab or lapatinib against HER2-positive resistant GC (105). Similarly, trastuzumab-resistant HER2-positive GC cells exhibit a circadian rhythm in glycolysis that is HK2-dependent. Regulated by the BMAL1–CLOCK–PER1–HK2 axis, the time-dependent co-administration of metformin enhances the efficacy of trastuzumab (106). This suggests that inhibiting glycolysis could serve as an adjunctive strategy to reverse resistance to targeted therapy. These findings provide mechanistic clues for PLGC research. Future research should investigate whether precancerous cells also develop similar glycolysis dependence and metabolic adaptations under prolonged intervention or therapeutic stress.

## 8 Discussion

Overall, glycolytic reprogramming in precancerous lesions of gastric cancer is unlikely to represent an isolated metabolic abnormality. Instead, available evidence suggests that it may connect chronic gastric mucosal injury, epithelial adaptation, abnormal proliferation, resistance to apoptosis, lactate transport, and local microenvironmental remodeling. Importantly, glycolytic activity may not increase continuously throughout the Correa cascade. Early Helicobacter pylori-associated inflammation, oxidative stress, and mitochondrial dysfunction may promote an adaptive glycolytic response, whereas extensive glandular loss during atrophy may be accompanied by a relative reduction in tissue-level metabolic activity. With the development of spasmolytic polypeptide-expressing metaplasia, intestinal metaplasia, and dysplasia, increased proliferative and biosynthetic demands may contribute to the reactivation of glycolysis. Thus, the proposed pattern of early activation, relative decline, and subsequent reactivation may reflect changes in the dominant cellular and pathological processes at different stages rather than a simple linear increase in glycolysis.

Some apparently inconsistent observations further indicate that glycolytic regulation in precancerous gastric lesions is dependent on pathological stage and biological context. For example, both increased and decreased TIGAR expression have been reported in different PLGC models. These differences may be related to the functional status of p53, the degree of oxidative stress, the pathological stage, or differences among experimental models. Similarly, changes in the expression of HIF-1α, glucose transporters, or glycolytic enzymes do not always correspond directly to changes in glycolytic flux, and measurements in bulk gastric tissues may also be affected by alterations in epithelial cell composition. Therefore, glycolysis-related findings should be interpreted together with pathological stage, cell type, experimental model, and metabolic endpoint (107). The consistent association among glycolysis-related regulation, pathological changes, proliferation and apoptosis, and responses to intervention supports the biological relevance of glycolytic reprogramming in PLGC, while also highlighting its stage-dependent and context-dependent characteristics.

## 9 Unresolved questions and future directions

Several issues remain to be addressed in studies of glycolytic reprogramming in precancerous lesions of gastric cancer. First, most available studies assess glycolytic alterations through changes in signaling molecules, metabolic enzymes, and metabolite levels. Although these findings provide important evidence of altered glucose metabolism, direct metabolic flux analyses remain relatively uncommon. Functional measurements of oxygen consumption rate, extracellular acidification rate, glycolytic rate, and glycolytic capacity have been reported in spasmolytic polypeptide-expressing metaplasia, but similar analyses across different pathological stages remain limited. The further application of stable-isotope tracing and quantitative metabolic flux analysis may help define glucose utilization more accurately during the progression of precancerous gastric lesions.

Current mechanistic evidence is derived predominantly from animal models, gastric epithelial cells, gastric organoids, and a limited number of human tissue studies. Further validation in larger and well-characterized human cohorts is needed to evaluate the consistency of glycolysis-related alterations among different populations and pathological stages. In addition, the stage-specific pattern described in this review is primarily based on cross-sectional comparisons. Longitudinal studies combining repeated endoscopic sampling with pathological and metabolic assessment would help clarify the temporal relationship between metabolic alterations and lesion progression.

Intervention studies have shown that pathological improvement is frequently accompanied by changes in glycolysis-related pathways and metabolites. However, many interventions, particularly traditional Chinese medicine formulations and natural bioactive compounds, regulate inflammation, oxidative stress, proliferation, apoptosis, autophagy, and metabolism simultaneously. Further studies using more specific metabolic inhibitors, genetic manipulation, and rescue experiments may help clarify the contribution of individual glycolytic targets to pathological progression and therapeutic responses. Emerging technologies may also provide important tools for investigating metabolic reprogramming in PLGC. Stable-isotope tracing and metabolic flux analysis can provide more direct information on glucose utilization and carbon flow. Spatial metabolomics and single-cell metabolomics may help characterize regional and cellular metabolic heterogeneity within gastric lesions, whereas patient-derived gastric organoids may provide a controllable model for investigating metabolic mechanisms and evaluating potential interventions. Together, metabolic flux analysis, human validation, longitudinal observation, emerging metabolic technologies, and more specific mechanistic studies will strengthen the evidence supporting glycolytic reprogramming as a potential biomarker and intervention target in precancerous lesions of gastric cancer. Addressing these questions will help clarify the stage-specific characteristics and biological significance of glycolytic reprogramming. It may also promote the development of risk assessment and metabolic intervention strategies for precancerous lesions of gastric cancer.

## Glossary

2-DG: 2-deoxy-D-glucose
5-FU: 5-fluorouracil
Akt: protein kinase B
AMPK: AMP-activated protein kinase
ATP: adenosine triphosphate
Bax: Bcl-2-associated X protein
Bcl-2: B-cell lymphoma 2
BMAL1: brain and muscle ARNT-like 1
BNIP3: BCL2-interacting protein 3
CAG: chronic atrophic gastritis
CagA: cytotoxin-associated gene A
CD147: cluster of differentiation 147
CDX2: caudal type homeobox 2
CDKN1C: cyclin-dependent kinase inhibitor 1C
ETC: electron transport chain
F2,6BP: fructose-2,6-bisphosphate
GC: gastric cancer
GES-1: gastric epithelial cell line 1
G6PDH: glucose-6-phosphate dehydrogenase
GLUT: glucose transporter
GLUT1: glucose transporter 1
GLUT3: glucose transporter 3
GLUT4: glucose transporter 4
GRg3: ginsenoside Rg3
GSH: glutathione
*H. pylori*: *Helicobacter pylori*
HER2: human epidermal growth factor receptor 2
HIF-1α: hypoxia-inducible factor 1 alpha
HK2: hexokinase 2
IM: intestinal metaplasia
LC3-II: microtubule-associated protein 1 light chain 3 II
LDH: lactate dehydrogenase
LDHA: lactate dehydrogenase A
LONP1: Lon protease 1
MCT1: monocarboxylate transporter 1
MCT4: monocarboxylate transporter 4
miRNA: microRNA
MNNG: N-methyl-N’-nitro-N-nitrosoguanidine
mTOR: mammalian target of rapamycin
NAD⁺: nicotinamide adenine dinucleotide
NADH: reduced nicotinamide adenine dinucleotide
NADP: nicotinamide adenine dinucleotide phosphate
NADPH: reduced nicotinamide adenine dinucleotide phosphate
OXPHOS: oxidative phosphorylation
PCNA: proliferating cell nuclear antigen
PDK1: pyruvate dehydrogenase kinase 1
PER1: period circadian regulator 1
PFK1: phosphofructokinase 1
PFK15: 6-phosphofructo-2-kinase/fructose-2,6-bisphosphatase 3 inhibitor 15
PFKFB3: 6-phosphofructo-2-kinase/fructose-2,6-bisphosphatase 3
PG I: pepsinogen I
PG II: pepsinogen II
PGR: pepsinogen I/II ratio
PI3K: phosphoinositide 3-kinase
PKM2: pyruvate kinase M2
PLGC: precancerous lesions of gastric cancer
PTEN: phosphatase and tensin homolog
ROS: reactive oxygen species
RSC: reserve stem cell
SD: Sprague-Dawley
SIRT3: sirtuin 3
Skp2: S-phase kinase-associated protein 2
SPEM: spasmolytic polypeptide-expressing metaplasia
TCA: tricarboxylic acid
TCM: traditional Chinese medicine
TIGAR: TP53-induced glycolysis and apoptosis regulator
TFF2: trefoil factor 2
TGF-β1: transforming growth factor beta 1
TNF-α: tumor necrosis factor alpha
TUNEL: terminal deoxynucleotidyl transferase dUTP nick end labeling
ULK1: unc-51-like autophagy activating kinase 1
VacA: vacuolating cytotoxin A
