# Supplementary material for: Glycolytic reprogramming in precancerous lesions of gastric cancer progression: pathogenesis and therapeutic potential
Source: Front Oncol. 2026 Aug 12;16:1885113. doi: 10.3389/fonc.2026.1885113 (PMC13507904; doi:10.3389/fonc.2026.1885113)
Supplement: Supplementary file 1 [file DataSheet1.docx]

Supplementary Material

# 1 Supplementary Data 1 Search strategies for PubMed and Web of Science

**Core search strategy for PubMed and Web of Science**

("gastric precancerous lesion*" OR "precancerous lesions of gastric cancer" OR "chronic atrophic gastritis" OR "intestinal metaplasia" OR "gastric dysplasia" OR "spasmolytic polypeptide-expressing metaplasia" OR SPEM) AND (glycolysis OR "glycolytic reprogramming" OR "glucose metabolism" OR "Warburg effect" OR lactate OR HIF-1alpha OR PI3K OR AKT OR mTOR OR GLUT1 OR GLUT4 OR HK2 OR PFKFB3 OR ENO1 OR PKM2 OR LDHA OR MCT1 OR MCT4 OR CD147 OR miR-34a OR miR-21 OR p53 OR TIGAR OR p57 OR CDKN1C)

**Supplementary search strategy for direct mechanistic evidence in gastric cancer**

("gastric precancerous lesion*" OR "precancerous lesions of gastric cancer" OR "chronic atrophic gastritis" OR "intestinal metaplasia" OR "gastric dysplasia" OR "spasmolytic polypeptide-expressing metaplasia" OR SPEM) AND (glycolysis OR "glycolytic reprogramming " OR "glucose metabolism" OR "Warburg effect" OR lactate OR HIF-1alpha OR PI3K OR AKT OR mTOR OR GLUT1 OR GLUT4 OR HK2 OR PFKFB3 OR ENO1 OR PKM2 OR LDHA OR MCT1 OR MCT4 OR CD147 OR miR-34a OR miR-21 OR p53 OR TIGAR OR p57 OR CDKN1C)

**Supplementary search strategy for indirect mechanistic evidence in gastric cancer**

("gastric cancer" OR "gastric epithelial cell*") AND (glycolysis OR "glucose metabolism" OR lactate) AND ("Helicobacter pylori" OR

HIF-1alpha OR PI3K OR AKT OR mTOR OR GLUT1 OR GLUT4 OR HK2 OR PFKFB3 OR PKM2 OR LDHA OR MCT1 OR MCT4 OR CD147 OR miR-34a OR miR-21 OR p53 OR TIGAR OR p57 OR CDKN1C)

# 2 Supplementary Tables

## 2.1 Supplementary Table 1. Evidence for Glycolytic Alterations and Mechanisms in PLGC

| **Evidence category** | **Major claim** | **Key molecular/metabolic findings** | **Evidence source and experimental model** | **Metabolic readout** | **Evidence level** | **Strengths and limitations** | **References** |
| --- | --- | --- | --- | --- | --- | --- | --- |
| **Stage-Specific Glycolytic Alterations** | The early paligenosis response may involve a temporary restraint on proliferation and metabolism during injury-induced metaplasia. | DDIT4-dependent signaling restricted mTORC1 activity and permitted proliferation preferentially in appropriately reprogrammed cells during injury-induced metaplasia. | Injury-induced gastric metaplasia models with mechanistic analyses of paligenosis. | Cell-state markers, proliferation, and mTORC1-related signaling; no direct glycolytic flux measurement. | E4 | **Strengths**  Mechanistic support for the atrophic transition  **Limitations**  Mechanistic extrapolation  No direct glycolytic flux analysis  No PLGC-specific validation | (25) |
|  | Chief-cell paligenosis is an mTORC1-dependent regenerative program that may alter energy demand during glandular injury. | Differentiated gastric cells re-entered the cell cycle through staged paligenosis controlled by mTORC1-dependent mechanisms. | Gastric injury and chief-cell paligenosis models. | Cell-state, mTORC1 activity, and regenerative proliferation; no glycolytic assay. | E4 | **Strengths**  Mechanistic support for stage-specific metabolic change  **Limitations**  No direct glycolytic flux analysis  No evidence of a transient glycolytic decline in PLGC | (26) |
|  | Glycolysis-related signaling is activated during early PLGC, particularly CAG. | Modified Zuojin pill reduced HIF-1α, GLUT1 and GLUT4, HK2, PFKFB3, PKM2, LDHA, glucose, pyruvate, lactate, and LDH; PCNA and Ki-67 decreased, whereas cleaved caspase-3 and TUNEL increased. | MNNG-induced PLGC in SD rats and MNNG-treated GES-1 gastric epithelial cells. | Protein and molecular expression; glucose, pyruvate, lactate, and LDH measurements; proliferation, apoptosis, and histopathology. | E2 | **Strengths**  PLGC-relevant preclinical evidence  Multiple metabolic endpoints  **Limitations**  Intervention-based study  No human validation  No longitudinal flux analysis  No isotope tracing | (27) |
|  | SPEM is associated with a shift from oxidative metabolism toward glycolysis regulated by p57. | Restoration of p57 reduced HIF-1α, LDHA, PKM2, pyruvate, lactate, glycolytic rate, and glycolytic capacity while suppressing SPEM and abnormal proliferation. | HDT-induced SPEM mice, TAM-treated GES-1 cells, and gastric organoids induced by MNNG or *H. pylori*. | Protein expression; pyruvate and lactate measurements; glycolytic rate and capacity; lineage and proliferation markers. | E2 | **Strengths**  Functional evidence from animal, epithelial cell, and organoid models  **Limitations**  No human SPEM validation  No longitudinal assessment of cancer progression | (28) |
| **Metabolic Regulation Associated with *H. pylori*** | Primary solid tumors can have slow TCA flux despite malignant growth. | Direct metabolic measurements showed slow TCA flux and ATP production in primary solid tumors compared with metastases. | Primary and metastatic solid tumors using in vivo metabolic analysis. | Isotope-based TCA flux and ATP production measurements. | E4 | **Strengths**  High-quality functional metabolic evidence  Isotope-based flux analysis  **Limitations**  Non-gastric evidence  Limited relevance to PLGC stage dynamics | (40) |
|  | Epigenetic intervention can reverse the Warburg effect and trigger mitochondrial cancer cell death. | Endogenous retroelement activation reversed glycolytic dependence and promoted mitochondria-mediated cell death. | Cancer cell and tumor models exposed to epigenetic therapy. | Glycolytic and mitochondrial function, cell death and therapeutic response. | E4 | **Strengths**  Mechanistic evidence of metabolic plasticity  **Limitations**  Non-gastric evidence  No PLGC validation | (41) |
|  | *H. pylori*-induced LONP1 contributes to mitochondrial stress and glycolytic reprogramming during gastric carcinogenesis. | *H. pylori* increased ATP-dependent Lon protease 1 and altered mitochondrial homeostasis and glycolysis-related phenotypes in gastric epithelial and gastric carcinogenesis models. | *H. pylori*-exposed gastric epithelial and gastric carcinogenesis experimental systems. | LONP1 expression, mitochondrial stress and glycolysis-related cellular endpoints. | E2 + E3 | **Strengths**  Relevant evidence for PLGC initiation  Mitochondrial and glycolysis-related endpoints  **Limitations**  No stage-resolved human PLGC analysis  No longitudinal glucose flux analysis | (42) |
|  | *H. pylori* activates PI3K/mTOR and increases HIF-1α in human gastric cells. | *H. pylori*-induced PI3K/mTOR activation increased HIF-1α and promoted loss of cyclin D1 with G0/G1 arrest. | Human gastric cell model infected with *H. pylori*. | PI3K/mTOR/HIF-1α signaling, cyclin D1, and cell cycle analysis; no direct glycolytic measurement. | E2 | **Strengths**  Relevant non-malignant gastric cell evidence  Upstream pathway analysis  **Limitations**  No direct glycolytic measurement  No human PLGC validation | (43) |
|  | CagA promotes SIRT3-related metabolic reprogramming and HIF-1α stabilization in GC. | CagA inhibited SIRT3-related signaling, increased ROS and HIF-1α stabilization, and altered PKM2/PDK1-associated metabolism. | GC models exposed to *H. pylori* CagA. | SIRT3, ROS, HIF-1α, and metabolism-related protein expression. | E3 | **Strengths**  Mechanistic evidence from GC models  **Limitations**  Indirect evidence for PLGC  No human PLGC validation | (45) |
|  | *H. pylori* disrupts mitochondrial import and mtDNA replication through VacA-dependent and independent mechanisms. | *H. pylori* targeted mitochondrial import machinery and mtDNA replication components, causing mitochondrial dysfunction. | *H. pylori* infection models with gastric epithelial cells and in vivo validation. | Mitochondrial protein import, mtDNA replication components, and mitochondrial injury; no glycolytic flux assay. | E2 | **Strengths**  Evidence of H. pylori-induced mitochondrial injury  **Limitations**  No direct glycolytic measurement  No stage-specific PLGC validation | (46) |
|  | VacA induces mitochondrial fission and host cell death. | VacA engaged mitochondrial fission machinery and caused mitochondrial fragmentation and cell death. | Cellular VacA exposure model. | Mitochondrial morphology, fission machinery and cell death. | E4 | **Strengths**  Mechanistic evidence of mitochondrial injury  **Limitations**  Non-PLGC-specific evidence  No direct glycolytic endpoint | (47) |
|  | VacA suppresses mTORC1 and reprograms host cell metabolism during *H. pylori* infection. | *H. pylori* altered host metabolism through VacA-dependent inhibition of mTORC1. | *H. pylori*-infected host cell models. | mTORC1 activity and host metabolic responses; no direct flux assessment at the PLGC stage. | E2 | **Strengths**  Relevant gastric infection model  **Limitations**  Direction of glycolytic change remains unclear  Duration of glycolytic change remains unclear  No PLGC-stage flux analysis | (48) |
|  | CagA enhances aerobic glycolysis and reduces 5-FU sensitivity in GC cells. | CagA increased glucose uptake, lactate production, p-Akt, HK2 and LDHA; glycolysis or Akt inhibition attenuated the phenotype. | CagA-overexpressing GC cell lines treated with 5-FU. | Glucose uptake, lactate production, protein expression, drug sensitivity and pathway inhibition. | E3 | **Strengths**  Functional evidence from GC cells  Pathway inhibition validation  **Limitations**  Indirect evidence for PLGC  No validation in precancerous human mucosa | (49) |
| **PI3K/Akt/mTOR and HIF-1α Signaling** | Intestinal metaplasia in *Atp4a*-deficient mice is accompanied by activation of proliferative and glycolysis-related signaling. | Strong p53 expression, increased Ki-67, activation of PI3K/Akt/mTOR/HIF-1α signaling and increased LDHA were reported during corpus intestinal metaplasia. | *Atp4a*−/− mouse model with dynamic characterization of corpus intestinal metaplasia. | Histological and molecular expression analyses; no direct glycolytic flux assay. | E2 | **Strengths**  Stage-relevant animal evidence  Dynamic pathological observation  **Limitations**  No functional metabolic assessment  No human longitudinal validation | (53) |
|  | The mTOR/HIF-1α axis contributes to glycolysis-related inflammation-associated carcinogenic transformation in CAG. | Hederagenin reduced mTOR/HIF-1α, HK2, PKM2, ENO1, and LDHA, decreased abnormalities in lactate production and glucose uptake, and improved gastric mucosal injury. | MNNG-induced CAG in SD rats and MNNG-treated gastric mucosal epithelial cells. | Protein expression, glucose uptake, lactate production, inflammatory markers, gastric function markers, and histopathology. | E2 | **Strengths**  PLGC-relevant functional preclinical evidence  **Limitations**  Pharmacological intervention study  No human validation  No rescue experiment  No isotope tracing | (54) |
| **GLUT-Mediated Glucose Uptake** | GLUT1, GLUT3, GLUT6, and GLUT10 are expressed in GC, and GLUT3 is associated with stage and survival. | Multiple glucose transporters were detected in human GC; GLUT3 correlated with UICC stage and survival. | Human GC tissue cohort. | Tissue expression and clinicopathological and survival associations. | E3 | **Strengths**  Direct human GC evidence  Clinicopathological and survival associations  **Limitations**  Indirect evidence for PLGC  Expression-based evidence  Timing of transporter upregulation remains unclear | (56) |
|  | A glycolysis-competent phenotype in GC is associated with poorer outcome. | Glycolytic competence in human GC negatively affected survival after paclitaxel-ramucirumab salvage treatment. | Clinical GC cohort. | Tumor glycolysis-related marker profiles and survival and treatment outcome analyses. | E3 | **Strengths**  Clinically relevant GC evidence  Treatment outcome data  **Limitations**  Indirect evidence for PLGC  Association-based evidence  No stage-specific PLGC data | (58) |
|  | GLUT expression increases across PLGC-related lesions and may contribute to progression and angiogenesis. | GLUT1 and GLUT4 were elevated in PLGC rats; GLUT1, GLUT3, and GLUT4 increased in human IM, dysplasia, and early GC tissues; Rg3 downregulated GLUT1 and GLUT4 and reduced angiogenesis-related changes. | Human IM, dysplasia, and early GC specimens plus a PLGC rat intervention model. | Immunohistochemical and protein expression, pathological staging, and angiogenesis-related endpoints. | E1 + E2 | **Strengths**  Direct human lesion evidence  Supportive animal evidence  **Limitations**  Expression-based evidence  No direct glucose flux analysis | (59) |
| **miRNA-Mediated Regulation** | miRNA-34a/LDHA and PI3K/Akt/mTOR/HIF-1α signaling are involved in glycolytic regulation in PLGC. | Weipixiao decoction increased miRNA-34a-related regulation; reduced LDHA, PI3K/Akt/mTOR/HIF-1α, MCT4, and CD147; and improved MNNG-induced PLGC. | MNNG-induced PLGC rats. | miRNA and protein expression, pathological endpoints, and lactate export-related proteins; no direct flux assay. | E2 | **Strengths**  PLGC-relevant animal evidence  **Limitations**  Intervention-based study  No direct target validation  No causal glycolytic validation | (63) |
|  | miRNA-21/PI3K/Akt signaling contributes to glycolysis-related remodeling in PLGC. | Ginsenoside Rg3 reduced miRNA-21 and PI3K/Akt/mTOR/HIF-1α signaling, decreased HK2 and LDHA, and altered apoptosis markers in *Atp4a*−/− mice. | *Atp4a*−/− PLGC mouse model with IM treated with ginsenoside Rg3. | miRNA and protein expression, apoptosis-related markers and pathological assessment; no direct flux assay. | E2 | **Strengths**  PLGC-relevant animal evidence  Pathway association  **Limitations**  No direct target validation  No functional glycolytic flux analysis | (65) |
| **p53/TIGAR and Redox Metabolism** | Pharmacological activation of wild-type p53 suppresses glycolytic enzymes. | Activated p53 inhibited glycolytic enzymes and reduced glycolysis-related cellular metabolism. | Non-PLGC cancer cell systems with pharmacological p53 activation. | Glycolytic enzyme expression and activity, together with cellular metabolic endpoints. | E4 | **Strengths**  Foundational p53 metabolic mechanism  **Limitations**  Mechanistic extrapolation  Dependent on wild-type p53  No PLGC-specific validation | (67) |
|  | TIGAR is a p53-inducible regulator that diverts glucose from glycolysis toward redox-supporting pathways. | TIGAR lowered fructose-2,6-bisphosphate/PFK1-driven glycolysis and supported the pentose phosphate pathway and redox homeostasis. | Foundational molecular and cellular models of p53/TIGAR biology. | Metabolic enzyme activity, glycolysis-related metabolites, and apoptosis and redox endpoints. | E4 | **Strengths**  Canonical TIGAR metabolic mechanism  **Limitations**  Non-gastric evidence  No PLGC-specific validation | (68) |
|  | The p53/miRNA-34a/LDHA axis regulates glycolysis and lactate export in PLGC. | Astragaloside IV increased p53/miRNA-34a-related signaling, decreased LDHA, HIF-1α, MCT1, MCT4 and CD147, and reversed MNNG-induced lesions. | MNNG-induced PLGC in rats. | miRNA and protein expression, lesion pathology, and lactate transporter markers. | E2 | **Strengths**  PLGC-relevant animal evidence  **Limitations**  Expression-based evidence  No lactate flux assessment  No extracellular pH measurement  No rescue experiment | (71) |
|  | TIGAR supports redox homeostasis and abnormal proliferation in PLGC. | Ginsenoside Rg3 reduced TIGAR, G6PDH, NADP and GSH, increased ROS and apoptosis, and decreased PCNA in PLGC rats. | Rat PLGC model treated with ginsenoside Rg3. | TIGAR and G6PDH expression; NADP, GSH, and ROS levels; apoptosis; and proliferation markers. | E2 | **Strengths**  PLGC-relevant metabolic and redox evidence  **Limitations**  Intervention-based study  No direct glycolytic flux analysis  No TP53 functional characterization | (72) |
| **p57-Mediated Regulation** | *CDKN1C* repression can promote aerobic glycolysis and proliferation in cancer cells. | The lncRNA BLACAT1 repressed *CDKN1C* through EZH2-induced H3K27me3 and increased proliferation, migration, and aerobic glycolysis in pancreatic cancer cells. | Pancreatic cancer cell models. | *CDKN1C* expression, epigenetic regulation, glucose and lactate measurements or aerobic glycolysis assays, and tumor cell phenotypes. | E4 | **Strengths**  Mechanistic evidence linking *CDKN1C* to glycolysis  **Limitations**  Non-gastric evidence  No validation across PLGC stages | (74) |
|  | p57 maintains the reserve stem cell state of gastric chief cells. | p57 maintained chief cell quiescence and reserve stem cell identity; loss of p57 enabled injury-responsive proliferation. | Mouse gastric chief cell lineage and injury and regeneration models. | Lineage tracing, p57 protein expression, cell state, and proliferation analyses; no direct glycolytic assay. | E2 | **Strengths**  Directly relevant gastric epithelial evidence  **Limitations**  No direct glycolytic assay  Evidence limited to cell-state regulation | (75) |
| **Imbalance Between Proliferation and Apoptosis** | NF-κB-related regulation of apoptosis contributes to reversal of PLGC. | Xiao Tan He Wei decoction improved MNNG-induced lesions and regulated apoptosis through the NF-κB pathway in vivo and in vitro. | MNNG-induced PLGC in animals and gastric cell models. | Apoptosis-related proteins, NF-κB signaling and histopathology; no direct glycolysis assay. | E2 | **Strengths**  PLGC-relevant pathological evidence  **Limitations**  No direct glycolytic assay  No causal validation | (77) |
|  | Mitochondrial ROS accumulation and STAT3 inhibition can alleviate PLGC. | (-)-Asarinin increased mitochondrial ROS, inhibited STAT3 signaling, and improved PLGC. | PLGC preclinical model. | Mitochondrial ROS, STAT3 signaling, apoptosis and proliferation, and pathology. | E2 | **Strengths**  PLGC-relevant redox and survival evidence  **Limitations**  No direct glycolytic evidence  No causal metabolic validation | (78) |
|  | *H. pylori* disrupts gastric epithelial homeostasis through macrophage-derived CCL3. | *H. pylori*-stimulated macrophages secreted CCL3, altering epithelial homeostasis and promoting pathogenic responses. | *H. pylori* infection models involving macrophage and epithelial interactions. | CCL3 secretion, immune cell signaling, and gastric epithelial outcomes. | E2 | **Strengths**  Relevant inflammatory-context evidence  **Limitations**  No direct glycolytic measurement  No causal metabolic validation | (80) |
|  | Proliferation, apoptosis, and p53 expression change across *H. pylori*-associated gastric epithelial lesions. | Human gastric epithelial lesions showed stage-associated changes in apoptosis, proliferation and p53 expression. | Human *H. pylori*-associated gastric epithelial lesions. | Histology, apoptosis, proliferation and p53 expression. | E1 | **Strengths**  Direct human lesion evidence  Stage-associated pathological data  **Limitations**  Older study  Non-metabolic evidence  No causal glycolytic validation | (82) |
|  | HIF-1α-mediated glycolysis is associated with an imbalance between proliferation and apoptosis during CAG and PLGC progression. | Modified Zuojin formula reduced HIF-1α, GLUT1 and GLUT4, HK2, PFKFB3, PKM2, LDHA and metabolites, while increasing apoptosis markers and improving pathology. | MNNG-induced gastric atrophy and PLGC rat model. | Glycolytic molecules and metabolites, apoptosis markers, inflammatory factors and histopathology. | E2 | **Strengths**  Integrated PLGC-relevant preclinical evidence  Metabolic and pathological endpoints  **Limitations**  Intervention-based study  No rescue experiment  No human validation  No causal validation | (88) |
| **Lactate Transport and Microenvironmental Remodeling** | CD147 is required for MCT1 and MCT4 membrane function and growth of glycolytic tumors. | CD147 supported MCT1 and MCT4 localization and lactate/H+ transport; disruption impaired tumor energetics and growth. | Glycolytic tumor cell and tumor growth models. | MCT localization, lactate/H+ transport, tumor energetics and growth. | E4 | **Strengths**  Strong mechanistic evidence for lactate export  **Limitations**  Non-gastric evidence  No PLGC-specific validation | (92) |
|  | MCT1, MCT4, and CD147 are associated with glycolysis and autophagy during PLGC progression. | Xiaojianzhong decoction reduced HIF-1α, MCT1, MCT4, CD147 and autophagy markers, increased SIRT6 and improved Ki-67/CDX2-related pathology. | MNNG-induced PLGC in Wistar rats. | Transporter and glycolysis-related protein expression, autophagy markers, proliferation and metaplasia, and histopathology. | E2 | **Strengths**  Directly relevant animal evidence  **Limitations**  No lactate concentration measurement  No lactate transport flux assessment  No extracellular acidification measurement  No rescue experiment | (94) |

Note: Evidence level: E1, direct evidence from human PLGC tissues or clinical samples; E2, evidence from PLGC-relevant animal models, gastric organoids, or non-malignant gastric epithelial models; E3, indirect evidence from GC tissues or experimental GC models; E4, extrapolated mechanistic evidence from other cancers, non-gastric systems, or foundational studies. The tier reflects relevance to PLGC rather than formal methodological quality. Reviews, guidelines, expert opinions, and meta-analyses were not assigned evidence tiers and were excluded from this study-level table. Changes in gene or protein expression were not considered equivalent to direct changes in glycolytic flux.

**Supplementary Table 2. Detailed botanical compositions of the traditional Chinese medicine formulations listed in Table 1**

| **Name** | **Composition or source** | **Reference** |
| --- | --- | --- |
| Modified Zuojin pill | *Astragalus Membranace us* Bunge [Fabaceae; Astragali Radix] (Huangqi)  Citrus reticulata Blanco [Rutaceae; Citri Reticulatae Pericarpium] (Chenpi)  *Panax notoginseng* (Burkill) F.H.Chen [Araliaceae; Notoginseng Radix et Rhizoma] (Sanqi)  *Coptis chinensis* Franch. [Ranunculaceae; Coptidis Rhizoma] (Huanglian)  *Tetradium ruticarpum* (A.Juss.) T.G.Hartley [Rutaceae; Evodiae Fructus] (Wuzhuyu)  *Glycyrrhiza uralensis* Fisch. ex DC. [Fabaceae; Glycyrrhizae Radix et Rhizoma] (Gancao) | (27) |
| Modified Zuojin formula | *Astragalus Membranace us* Bunge [Fabaceae; Astragali Radix] (Huangqi)  *Citrus reticulata* Blanco [Rutaceae; Citri Reticulatae Pericarpium] (Chenpi)  *Panax notoginseng* (Burkill) F.H.Chen [Araliaceae; Notoginseng Radix et Rhizoma] (Sanqi)  *Glycyrrhiza uralensis* Fisch. ex DC. [Fabaceae; Glycyrrhizae Radix et Rhizoma] (Gancao) | (88) |
| Xiaojianzhong decoction | Maltose（Yitang）  *Cinnamomum cassia* (L.) J.Presl [Lauraceae;Cinnamomi Ramulus] (Guizhi)  *Paeonia lactiflora* Pall. [Paeoniaceae; Paeoniae Radix Alba] (Baishao)  *Zingiber officinale* Roscoe [Zingiberaceae; Zingiberis Rhizoma Recens] (Shengjiang)  *Glycyrrhiza uralensis* Fisch. ex DC. [Fabaceae; Glycyrrhizae Radix et Rhizoma] (Gancao)  *Ziziphus jujuba* Mill. [Rhamnaceae; Jujubae Fructus] (Dazao) | (94) |
| Weipixiao decoction | *Astragalus Membranace us* Bunge [Fabaceae; Astragali Radix] (Huangqi)  *Pseudostellaria heterophylla* (Miq.) Pax [Caryophyllaceae; Pseudostellariae Radix] (Taizishen)  *Atractylodes macrocephala* Koidz. [Asteraceae; Atractylodis Macrocephalae Rhizoma] (Baizhu)  *Salvia miltiorrhiza* Bunge [Lamiaceae; Salviae Miltiorrhizae Radix et Rhizoma] (Danshen)  *Hedyotis diffusa* Willd. [Rubiaceae; Hedyotis Diffusae Herba] (Baihua Sheshecao) | (63) |
| Pristimerin | *Celastrus orbiculatus* Thunb. [Celastraceae; Celastri Orbiculati Caulis] (Nansheteng) | (28) |
| Hederagenin | Active components screened from Jianwei Xiaoyan Granule formula by molecular docking and SPR | (54) |
| Ginsenoside Rg3 | *Panax ginseng* C.A. Meyer [Araliaceae; Ginseng Radix et Rhizoma] (Renshen) | (65) |
| Astragaloside IV | *Astragalus Membranace us* Bunge [Fabaceae; Astragali Radix] (Huangqi) | (71) |

**References**

25.	Miao Z-F, Sun J-X, Adkins-Threats M, Pang M-J, Zhao J-H, Wang X, Tang K-W, Wang Z-N, Mills JC. DDIT4 Licenses Only Healthy Cells to Proliferate During Injury-induced Metaplasia. *Gastroenterology* (2021) 160:260-271.e10. doi: 10.1053/j.gastro.2020.09.016

26.	Willet SG, Lewis MA, Miao Z-F, Liu D, Radyk MD, Cunningham RL, Burclaff J, Sibbel G, Lo H-YG, Blanc V, et al. Regenerative proliferation of differentiated cells by mTORC1-dependent paligenosis. *EMBO J* (2018) 37:e98311. doi: 10.15252/embj.201798311

27.	Liu S, Ji H, Zhang T, Huang J, Yin X, Zhang J, Wang P, Wang F, Tang X. Modified Zuojin pill alleviates gastric precancerous lesions by inhibiting glycolysis through the HIF-1α pathway. *Phytomedicine* (2025) 136:156255. doi: 10.1016/j.phymed.2024.156255

28.	Wen J-S, Pan Z-W, Yao X-D, Liu Y-Q, Zhu Y-D. Pristimerin ameliorates spasmolytic polypeptide expressing metaplasia by modulating Cdkn1c (p57)-mediated glycolytic reprogramming. *World J Gastroenterol* (2026) 32:113771. doi: 10.3748/wjg.v32.i10.113771

40.	Bartman CR, Weilandt DR, Shen Y, Lee WD, Han Y, TeSlaa T, Jankowski CSR, Samarah L, Park NR, da Silva-Diz V, et al. Slow TCA flux and ATP production in primary solid tumours but not metastases. *Nature* (2023) 614:349–357. doi: 10.1038/s41586-022-05661-6

41.	Fresquet V, Garcia-Barchino MJ, Larrayoz M, Celay J, Vicente C, Fernandez-Galilea M, Larrayoz MJ, Calasanz MJ, Panizo C, Junza A, et al. Endogenous Retroelement Activation by Epigenetic Therapy Reverses the Warburg Effect and Elicits Mitochondrial-Mediated Cancer Cell Death. *Cancer Discov* (2021) 11:1268–1285. doi: 10.1158/2159-8290.CD-20-1065

42.	Luo B, Wang M, Hou N, Hu X, Jia G, Qin X, Zuo X, Liu Y, Luo K, Song W, et al. ATP-Dependent Lon Protease Contributes to Helicobacter pylori-Induced Gastric Carcinogenesis. *Neoplasia* (2016) 18:242–252. doi: 10.1016/j.neo.2016.03.001

43.	Canales J, Valenzuela M, Bravo J, Cerda-Opazo P, Jorquera C, Toledo H, Bravo D, Quest AFG. Helicobacter pylori Induced Phosphatidylinositol-3-OH Kinase/mTOR Activation Increases Hypoxia Inducible Factor-1α to Promote Loss of Cyclin D1 and G0/G1 Cell Cycle Arrest in Human Gastric Cells. *Front Cell Infect Microbiol* (2017) 7:92. doi: 10.3389/fcimb.2017.00092

45.	Lee DY, Jung DE, Yu SS, Lee YS, Choi BK, Lee YC. Regulation of SIRT3 signal related metabolic reprogramming in gastric cancer by Helicobacter pylori oncoprotein CagA. *Oncotarget* (2017) 8:78365–78378. doi: 10.18632/oncotarget.18695

46.	Chatre L, Fernandes J, Michel V, Fiette L, Avé P, Arena G, Jain U, Haas R, Wang TC, Ricchetti M, et al. Helicobacter pylori targets mitochondrial import and components of mitochondrial DNA replication machinery through an alternative VacA-dependent and a VacA-independent mechanisms. *Sci Rep* (2017) 7:15901. doi: 10.1038/s41598-017-15567-3

47.	Jain P, Luo Z-Q, Blanke SR. Helicobacter pylori vacuolating cytotoxin A (VacA) engages the mitochondrial fission machinery to induce host cell death. *Proc Natl Acad Sci U S A* (2011) 108:16032–16037. doi: 10.1073/pnas.1105175108

48.	Kim I-J, Lee J, Oh SJ, Yoon M-S, Jang S-S, Holland RL, Reno ML, Hamad MN, Maeda T, Chung HJ, et al. Helicobacter pylori Infection Modulates Host Cell Metabolism through VacA-Dependent Inhibition of mTORC1. *Cell Host Microbe* (2018) 23:583-593.e8. doi: 10.1016/j.chom.2018.04.006

49.	Gao S, Song D, Liu Y, Yan H, Chen X. Helicobacter pylori CagA Protein Attenuates 5-Fu Sensitivity of Gastric Cancer Cells Through Upregulating Cellular Glucose Metabolism. *Onco Targets Ther* (2020) 13:6339–6349. doi: 10.2147/OTT.S230875

53.	Liu W, Yang L-J, Liu Y-L, Yuan D-S, Zhao Z-M, Wang Q, Yan Y, Pan H-F. Dynamic characterization of intestinal metaplasia in the gastric corpus mucosa of Atp4a-deficient mice. *Biosci Rep* (2020) 40:BSR20181881. doi: 10.1042/BSR20181881

54.	Liu J, Chen Y, Li M, Chen G, Yang J, Jiang Y, Zhang X, Li F, Hua H. Hederagenin suppresses inflammation-cancer transformation in chronic atrophic gastritis by modulating glycolysis through the mTOR/HIF-1α axis. *Eur J Pharmacol* (2026) 1021:178835. doi: 10.1016/j.ejphar.2026.178835

56.	Schlößer HA, Drebber U, Urbanski A, Haase S, Baltin C, Berlth F, Neiß S, von Bergwelt-Baildon M, Fetzner UK, Warnecke-Eberz U, et al. Glucose transporters 1, 3, 6, and 10 are expressed in gastric cancer and glucose transporter 3 is associated with UICC stage and survival. *Gastric Cancer* (2017) 20:83–91. doi: 10.1007/s10120-015-0577-x

58.	Ruzzo A, Graziano F, Bagaloni I, Di Bartolomeo M, Prisciandaro M, Aprile G, Ongaro E, Vincenzi B, Perrone G, Santini D, et al. Glycolytic competence in gastric adenocarcinomas negatively impacts survival outcomes of patients treated with salvage paclitaxel-ramucirumab. *Gastric Cancer* (2020) 23:1064–1074. doi: 10.1007/s10120-020-01078-0

59.	Zeng Z, Nian Q, Chen N, Zhao M, Zheng Q, Zhang G, Zhao Z, Chen Y, Wang J, Zeng J, et al. Ginsenoside Rg3 inhibits angiogenesis in gastric precancerous lesions through downregulation of Glut1 and Glut4. *Biomedicine & Pharmacotherapy* (2022) 145:112086. doi: 10.1016/j.biopha.2021.112086

63.	Cai T, Zhang C, Zeng X, Zhao Z, Yan Y, Yu X, Wu L, Lin L, Pan H. Protective effects of Weipixiao decoction against MNNG-induced gastric precancerous lesions in rats. *Biomed Pharmacother* (2019) 120:109427. doi: 10.1016/j.biopha.2019.109427

65.	Liu W, Pan H-F, Yang L-J, Zhao Z-M, Yuan D-S, Liu Y-L, Lin L-Z. Panax ginseng C.A. Meyer (Rg3) Ameliorates Gastric Precancerous Lesions in Atp4a-/- Mice via Inhibition of Glycolysis through PI3K/AKT/miRNA-21 Pathway. *Evid Based Complement Alternat Med* (2020) 2020:2672648. doi: 10.1155/2020/2672648

67.	Zawacka-Pankau J, Grinkevich VV, Hünten S, Nikulenkov F, Gluch A, Li H, Enge M, Kel A, Selivanova G. Inhibition of Glycolytic Enzymes Mediated by Pharmacologically Activated p53. *Journal of Biological Chemistry* (2011) 286:41600–41615. doi: 10.1074/jbc.M111.240812

68.	Bensaad K, Tsuruta A, Selak MA, Vidal MNC, Nakano K, Bartrons R, Gottlieb E, Vousden KH. TIGAR, a p53-inducible regulator of glycolysis and apoptosis. *Cell* (2006) 126:107–120. doi: 10.1016/j.cell.2006.05.036

71.	Zhang C, Cai T, Zeng X, Cai D, Chen Y, Huang X, Gan H, Zhuo J, Zhao Z, Pan H, et al. Astragaloside IV reverses MNNG-induced precancerous lesions of gastric carcinoma in rats: Regulation on glycolysis through miRNA-34a/LDHA pathway. *Phytother Res* (2018) 32:1364–1372. doi: 10.1002/ptr.6070

72.	Lv S, Chen X, Chen Y, Gong D, Mao G, Shen C, Xia T, Cheng J, Luo Z, Cheng Y, et al. Ginsenoside Rg3 induces apoptosis and inhibits proliferation by down-regulating TIGAR in rats with gastric precancerous lesions. *BMC Complement Med Ther* (2022) 22:188. doi: 10.1186/s12906-022-03669-z

74.	Zhou X, Gao W, Hua H, Ji Z. LncRNA-BLACAT1 Facilitates Proliferation, Migration and Aerobic Glycolysis of Pancreatic Cancer Cells by Repressing CDKN1C via EZH2-Induced H3K27me3. *Front Oncol* (2020) 10: doi: 10.3389/fonc.2020.539805

75.	Lee J-H, Kim S, Han S, Min J, Caldwell B, Bamford A-D, Rocha ASB, Park J, Lee S, Wu S-HS, et al. p57Kip2 imposes the reserve stem cell state of gastric chief cells. *Cell Stem Cell* (2022) 29:826-839.e9. doi: 10.1016/j.stem.2022.04.001

77.	Xu J, Shen W, Pei B, Wang X, Sun D, Li Y, Xiu L, Liu X, Lu Y, Zhang X, et al. Xiao Tan He Wei Decoction reverses MNNG-induced precancerous lesions of gastric carcinoma in vivo and vitro: Regulation of apoptosis through NF-κB pathway. *Biomedicine & Pharmacotherapy* (2018) 108:95–102. doi: 10.1016/j.biopha.2018.09.012

78.	Zhao M, Wen Y, Yang Y, Pan H, Xie S, Shen C, Liao W, Chen N, Zheng Q, Zhang G, et al. (-)-Asarinin alleviates gastric precancerous lesions by promoting mitochondrial ROS accumulation and inhibiting the STAT3 signaling pathway. *Phytomedicine* (2024) 126:155348. doi: 10.1016/j.phymed.2024.155348

80.	Wei Y-F, Li X, Zhao M-R, Liu S, Min L, Zhu S-T, Zhang S-T, Xie S-A. Helicobacter pylori disrupts gastric mucosal homeostasis by stimulating macrophages to secrete CCL3. *Cell Commun Signal* (2024) 22:263. doi: 10.1186/s12964-024-01627-5

82.	Zhang Z, Yuan Y, Gao H, Dong M, Wang L, Gong Y-H. Apoptosis, proliferation and p53 gene expression of H. pylori associated gastric epithelial lesions. *World J Gastroenterol* (2001) 7:779–782. doi: 10.3748/wjg.v7.i6.779

88.	Liu S, Zhang T, Fang L, Hu L, Yin X, Tang X. Integrative pharmacological analysis of modified Zuojin formula: Inhibiting the HIF-1α-mediated glycolytic pathway in chronic atrophic gastritis. *J Ethnopharmacol* (2025) 339:119136. doi: 10.1016/j.jep.2024.119136

92.	Le Floch R, Chiche J, Marchiq I, Naiken T, Ilc K, Murray CM, Critchlow SE, Roux D, Simon M-P, Pouysségur J. CD147 subunit of lactate/H+ symporters MCT1 and hypoxia-inducible MCT4 is critical for energetics and growth of glycolytic tumors. *Proc Natl Acad Sci U S A* (2011) 108:16663–16668. doi: 10.1073/pnas.1106123108

94.	Zhang J-X, Bao S-C, Chen J, Chen T, Wei H-L, Zhou X-Y, Li J-T, Yan S-G. Xiaojianzhong decoction prevents gastric precancerous lesions in rats by inhibiting autophagy and glycolysis in gastric mucosal cells. *World J Gastrointest Oncol* (2023) 15:464–489. doi: 10.4251/wjgo.v15.i3.464
